# Reduced Worries of Hypoglycaemia, High Satisfaction, and Increased Perceived Ease of Use after Experiencing Four Nights of MD-Logic Artificial Pancreas at Home (DREAM4)

**DOI:** 10.1155/2015/590308

**Published:** 2015-10-25

**Authors:** Claudia Ziegler, Alon Liberman, Revital Nimri, Ido Muller, Simona Klemenčič, Nataša Bratina, Sarah Bläsig, Kerstin Remus, Moshe Phillip, Tadej Battelino, Olga Kordonouri, Thomas Danne, Karin Lange

**Affiliations:** ^1^Diabetes Centre for Children and Adolescents, Kinder- und Jugendkrankenhaus AUF DER BULT, 30173 Hannover, Germany; ^2^The Jesse Z and Sara Lea Shafer Institute for Endocrinology and Diabetes, National Center for Childhood Diabetes, Schneider Children's Medical Center of Israel, 49202 Petah Tikva, Israel; ^3^Department of Pediatric Endocrinology, Diabetes and Metabolism, University Medical Centre-University Children's Hospital, 1000 Ljubljana, Slovenia; ^4^Department of Medical Psychology, Hannover Medical School, 30625 Hannover, Germany

## Abstract

*Aims.* This study assesses the impact of using an AP-system at home on fear of hypoglycaemia. In addition, satisfaction and acceptance of the new technology are evaluated. *Methods.* In a multicentre, multinational study of 75 patients using the MD-Logic AP during four consecutive nights in home setting 59 of them (aged 10–54 years, 54% male, HbA1c 7.89 ± 0.69% [62.72 ± 7.51 mmol/mol], diabetes duration 11.6 ± 8.4 yrs) answered standardized questionnaires (HFS, adapted TAM, and AP satisfaction) before and after using the AP. *Results.* After experiencing the AP in home setting worries of hypoglycaemia were significantly reduced (before 1.04 ± 0.53 versus after 0.90 ± 0.63; *P* = 0.017). Perceived ease of use as a measure of acceptance with the AP significantly increased after personal experience (before 4.64 ± 0.94 versus after 5.06 ± 1.09; *P* = 0.002). The overall satisfaction mean score after using the AP was 3.02 ± 0.54 (range 0–4), demonstrating a high level of satisfaction with this technology. *Conclusions.* The four-night home-based experience of using MD Logic AP was associated with reduced worries of hypoglycaemia, high level of satisfaction, and increased perceived ease of use of the new technology in children, adolescents, and adults.

## 1. Introduction

Current research is focusing on the artificial pancreas (AP) or the so-called closed-loop systems (CLS) to optimize metabolic control in patients with type 1 diabetes mellitus (T1DM). An AP combines continuous subcutaneous insulin infusion (CSII) and continuous glucose monitoring (CGM) with a control algorithm to calculate insulin delivery in response to sensor data. Different artificial pancreas systems from various research groups have shown the superiority of the artificial pancreas compared to standard CSII therapy regarding overall glucose control and risk of nocturnal hypoglycaemia. These results were achieved during controlled conditions of an inpatient environment (review by [1]). Now first studies started to evaluate the AP system at the patient's home. The Diabetes Wireless Artificial Pancreas Consortium (DREAM-Project, [2]) assessed the MD-Logic artificial pancreas system [3, 4] outside hospital settings under real-life conditions. Meanwhile the safety and efficacy of the MD-Logic automated insulin delivery system was demonstrated in hospital setting [1] as well as in diabetes camps [2] and in home setting [5, 6]. Briefly, The MD-Logic is a wireless fully automated closed-loop system based on a fuzzy logic theory algorithm, a learning algorithm, a personalized system setting, and alerts module. The alerts module includes real-time alarms such as impending hypoglycaemia and long standing hyperglycaemia. The algorithm for alerts integrates information derived from past glucose levels and insulin delivery (time and dose) as well as models of insulin pharmacodynamics. The hypoglycaemia alarms are designed to operate in instances when impending hypoglycaemia cannot be avoided by holding insulin alone [4].

While the metabolic efficacy of the existing AP systems is impressing, its psychological impact remains to be evaluated. The majority of patients accept and use CSII continuously, but there are also reports of some patients who discontinued this technology [7, 8]. CGM use was less effective in adolescents due to the low rate of young people willing to use CGM continuously [9]. Barriers mentioned against CGM use were, for example, “technical aspects” like alarms and inaccurate readings and “body image concerns to wear two devices” [10]. Barriers like fear of hypoglycaemia and human factors like the emotional acceptance of wearing the devices and trusting the accuracy seem to play a leading part for the acceptance and efficacy of these technologies.

Until now the acceptance of CLS was rarely assessed. Elleri and colleagues [11] prospectively asked parents of children with T1DM if they would trust an AP-system. The majority (90%) reported secure feelings. A sample of 132 adults with T1DM also indicated positive attitudes towards the new technology [12]. However, these patients and parents had no real-life access to the system.

Little is known on the psychological impact of an AP system in the patient's home [13]. Systematic studies on fear of hypoglycaemia and satisfaction with and acceptance of an AP in children, adolescents, and adults with T1DM in the home setting have not yet been evaluated. In this study the impact of using an AP-system during four consecutive nights in a home setting regarding fear of hypoglycaemia is assessed among children, adolescents, and adults in a multicentre study. In addition, satisfaction and acceptance of this new technology are evaluated as main psychological predictors of a potential long-term use of the AP system.

## 2. Materials and Methods

### 2.1. Trial Design

This study on the psychological impact of using an AP for four consecutive nights in home setting is part of the DREAM Project (DREAM4) conducted in three multinational centres from Israel, Slovenia, and Germany. The main study focused on the feasibility, safety, and efficacy of the MD-Logic AP. It is a two-arm study, each arm covering four consecutive nights comparing the MD-Logic AP (“closed-loop” arm) with sensor-augmented pump therapy (“control” arm). Patients were randomly assigned either to “Group A” (first “closed-loop” and then “control” arm) or to “Group B” (vice versa) with a week washout between the two periods [5, 6]. Before intervention and after experiencing the MD-Logic AP participants answered structured questionnaires on fear of hypoglycaemia, satisfaction with the technology, and acceptance of the MD-Logic AP. The study was conducted in compliance with the protocol, the Declaration of Helsinki, and applicable regulatory and good clinical practice requirements. All patients and parents provided a written informed consent prior to trial initiation.

### 2.2. Participants and Eligibility Criteria

Overall 45 patients from the Schneider Children's Medical Center in Israel (Petah Tikva), 15 patients from the University Medical Centre-University Children's Hospital in Slovenia (Ljubljana), and 15 patients from the Kinder- und Jugendkrankenhaus AUF DER BULT in Germany (Hanover) were recruited between November 2012 and January 2014. Main inclusion criteria were type 1 diabetes (>1 yr since diagnosis), age ≥10 years and ≤65 years, CSII therapy for at least three months, experience in using CGM, HbA_1c_ ≥7% to <10% (53–86 mmol/mol), patients living with at least one other adult person, and an internet access at patient's home. Main exclusion criteria were concomitant diseases that influence metabolic control, participation in any other study, pregnancy, a history of diabetic ketoacidosis or severe hypoglycaemia within the last month, medications, or other conditions that may influence metabolic control, compromise safety, or prevent subjects from completing the study [5, 6]. For organisational reasons sixty participants were offered to answer psychological questionnaires, 30 from Israel, 15 from Slovenia, and 15 from Germany.

### 2.3. Psychological Assessment

*Fear of Hypoglycaemia.* Fear of hypoglycaemia was assessed with the Hypoglycaemia Fear Survey (HFS). The HFS is based on cognitive-behavioural theory of anxiety distinguishing emotional and behavioural components. Accordingly the HFS includes a Behaviour Subscale (HFS-B) and a Worry Subscale (HFS-W). The HFS adult-version consists of 10 behaviour or avoidance items (items 1–10) and 17 worry or affect items (items 11–27) to be answered on a 5-point Likert scale. Higher total scores reflect greater fear of hypoglycaemia. Higher scores on the behaviour subscale reflect a greater tendency to avoid hypoglycaemia and/or its negative consequences. Higher scores on the Worry Subscale indicate more worries concerning episodes of hypoglycaemia and its consequences. A study with 158 individuals with type 1 diabetes indicated good internal reliability: Cronbach's alpha for the entire scale was .90, for the Behaviour Subscale .60, and for the Worry Subscale .89. The instrument proved to have a high test-retest stability (after 6 weeks .89, .81, and .85 (*P* < 0.001)) and a good construct validity as the HFS covaries with elevated HbA_1c_ and is sensitive to a hypoglycaemia awareness training [14].

The HFS was adapted to be answered by children with T1DM. The final pediatric HFS (C-HFS) questionnaire consists of 10 behaviour or avoidance items (items 1–10) and 15 worry or affect items (items 11–25) to be answered on a 5-point Likert scale. A study with adolescents demonstrated adequate internal consistency for the C-HFS-Total Score and the C-HFS-W Score (.86, .91), with a lower Cronbach's alpha for the C-HFS-B Score (.54) [15]. Green reported similar results [16]. Construct validity was demonstrated by a significant correlation between State-trait Anxiety Inventory for children scores and C-HFS-Total scores and C-HFS-W Scores [15]. 

*Acceptance of the Artificial Pancreas.* The acceptance of an artificial pancreas was assessed with the adapted TAM Questionnaire. This instrument developed by van Bon et al. [12] is based on the Technology Acceptance Model (TAM). The questionnaire consists of two items assessing “Intention to Use” (items 1-2), eight items on “Perceived Usefulness” and its determinants (items 3–10), three items on “Perceived Ease of Use” (items 11–13), and one item on “Trust” (item 14). The items are answered on a 7-point Likert scale. Higher scores indicate a higher degree of acceptance of the AP. In a study with 132 patients with T1DM Cronbach's alpha was .91, reflecting a good internal consistency.

*Satisfaction with Use of an Artificial Pancreas.* The questionnaire was developed and validated specifically for closed-loop studies [17]. The questionnaire consists of 14 items (e.g., item 1: “in general to which extent were you satisfied with using the artificial pancreas system?”). Items are answered on a 5-point Likert scale. A higher score indicates a higher degree of satisfaction with the AP.

All questionnaires were translated linguistically in patients' native language; the validation of the translation was performed by each study centre.

Sociodemographic characteristics (age, gender, and family status) and clinical characteristics (HbA_1c_, onset of diabetes, start of CSII, and sensor use) were collected from patients' files. Metabolic control was assessed by DCA 2000 in all centres.

### 2.4. Statistical Methods

All analyses were performed with SPSS for Windows version 22. The descriptive statistics are reported as percentages or means and standard deviations (SD). Comparison between pre- and postassessment was performed using paired Fisher-*t*-test or Wilcoxon signed-rank test. Effects of age-group, gender, or regular sensor use were analysed by using ANOVA or Kruskal-Wallis *H* test. Associations between fear of hypoglycaemia, acceptance, satisfaction and HbA_1c_, diabetes duration, and pump duration were calculated via Spearman's rho. Cronbach's alpha was performed by analyses of reliability. Varimax rotated factor analyses were applied to assess the structure of the questionnaires. Two-sided *P* values ≤ 0.05 were considered statistically significant.

## 3. Results

### 3.1. Study Sample

Overall 59 patients (54% male, age 19.9 ± 9.9 yrs, diabetes duration 11.6 ± 8.4 yrs, HbA_1c_ 7.89 ± 0.69% [62.7 ± 7.5 mmol/mol]) answered the questionnaires before and after using the AP for four consecutive nights at home (29 patients from Israel, 15 from Slovenia, and 15 from Germany). One additional patient withdrew consent. Baseline demographic and diabetes characteristics were similar over centres. CGM use turned out to differ between centres (Table 1). Patients from Germany had previous continuous CGM use less often (3 versus 12) compared to Israel (18 versus 11) or Slovenia (8 versus 7). Overall, significantly fewer adult than younger patients had used the device continuously at baseline (Table 1).

### 3.2. Fear of Hypoglycaemia

This questionnaire was completed by 58 participants.

*Internal Consistency.* For the total scale of the adult version Cronbach's alpha was .88, suggesting a high level of reliability. The Behaviour Subscale had an alpha of .61, and the Worry Subscale had an alpha of .90, comparable to the results published by Cox and colleagues [14]. Cronbach's alpha for the pediatric version was .69, demonstrating an adequate reliability. As reported elsewhere the Behaviour Subscale shows consistently a lower internal consistency [15, 16].

**Figure 1:**
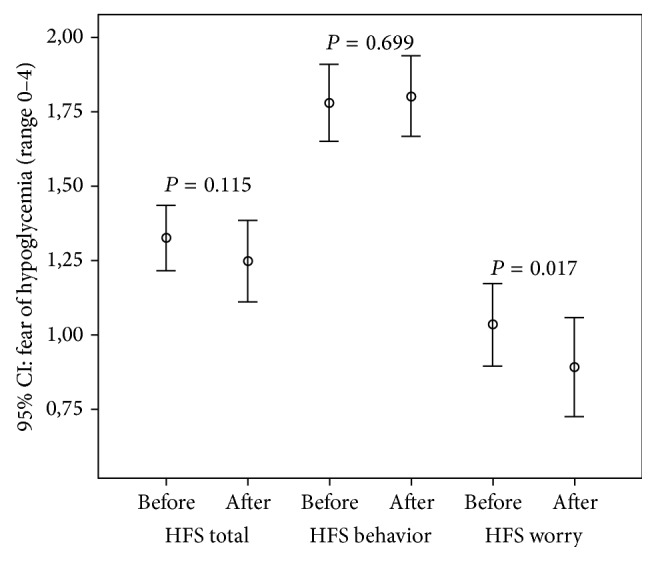
Hypoglycaemia Fear Survey (HFS) before and after 4 nights with the MD-Logic artificial pancreas in home setting (*n* = 58).

At study entry overall HFS items' mean score was 1.33 ± 0.41; for the Behaviour Subscale it was 1.78 ± 0.49 and for the Worry Subscale was 1.04 ± 0.53 (range 0–4). After four nights on the AP, the HFS Worry Score decreased (1.04 ± 0.53 versus 0.90 ± 0.63; *P* = 0.017). The HFS Total Score and HFS Behaviour Score remained on a low level of anxiety (Figure 1).

There were no significant differences among all HFS scales at study entry or at follow-up in relation to the patients' demographic or diabetes characteristics (each *P* > 0.1).

### 3.3. Acceptance of the Artificial Pancreas

55 patients answered all items of the TAM questionnaire.

*Internal Consistency.* For the total scale Cronbach's alpha was .90, reflecting a good internal consistency. The “Intention to Use” subscale had an alpha of .83 and the “Perceived Usefulness” subscale revealed a Cronbach's alpha of .87, reflecting an adequate reliability. The “Perceived Ease of Use” subscale had an alpha of .71.

*Factor Analysis.* The principal components analysis revealed the presence of four components with eigenvalues exceeding 1, explaining 43.7%, 9.8%, 8.6%, and 7.2% of the variance.

The overall TAM score at study entry was 4.69 ± 0.87; for the “Intention to Use” subscale it was 4.75 ± 1.25, for the “Perceived Usefulness” subscale was 4.66 ± 0.91, for the “Perceived Ease of Use” subscale was 4.64 ± 0.94, and for the “Trust” item was 4.86 ± 1.24 (range each 0–6). After four nights on AP at home the “Perceived Ease of Use” score increased (4.64 ± 0.94 versus 5.06 ± 1.09; *P* = 0.002). The other subscales of TAM remained on a high level (Table 2) with no significant association to age, diabetes duration, gender, or metabolic control (each *P* > 0.1).

Patients using CGM continuously reported a higher acceptance of AP on all TAM scales compared to those with no regular use (Table 3). Accordingly there were significant centre-differences on TAM “Intention to Use” subscale (*P* = 0.032) and TAM “Perceived Usefulness” subscale (*P* = 0.038) with lower scores in the German sample compared to the ones from Slovenia and Israel.

### 3.4. Satisfaction

The satisfaction questionnaire was completed by 57 patients. 

*Factor Analysis.* The principal components analysis revealed the presence of five components with eigenvalues exceeding 1, explaining 34.47%, 11.59%, 9.98%, 8.08%, and 7.47% of variance. Every item has only one high correlation with one factor, ranging from .56 to .87. Five scales can be identified: scale 1 “Perceived Usefulness of Alarms” (items 8, 9, and 12); scale 2 “Trust” (items 2, 6, and 11), scale 3 “Ease of Use” (items 3, 5, and 7); scale 4 “Satisfaction” (items 1, 13, and 14), and scale 5 “Freedom” (items 4, 10).

*Internal Consistency*. For the total scale Cronbach's alpha was .84, reflecting a good internal consistency. Only if item 10 would have been deleted there is an increase in Cronbach's alpha to .85. The “Perceived Usefulness of Alarms” subscale showed an alpha of .75, “Trust” subscale had an alpha of .73, “Ease of Use” subscale had an alpha of .72, “Satisfaction” subscale had an alpha of .77, and “Freedom” subscale had an alpha of .56 reflecting a lower internal consistency.

The overall satisfaction score was 3.02 ± 0.54; for “Perceived Usefulness of Alarms” subscale it was 2.82 ± 0.77, for “Trust” subscale was 3.07 ± 0.79, for “Ease of Use” subscale was 3.26 ± 0.73, for “Satisfaction” subscale was 3.16 ± 0.77, and for “Freedom” subscale was 2.66 ± 0.91 (range 0–4). There were significant differences of “Ease of Use” subscale (*P* = 0.004) between the age groups with significant lower mean scores in children than in adolescents/adults (Table 4).

Significant differences of overall satisfaction mean score, “Perceived Usefulness of Alarms” subscale, “Satisfaction” subscale, and “Freedom” subscale according to regular sensor use with significant higher mean scores in patients using CGM continuously were observed (*P* = 0.001, *P* = 0.002, *P* = 0.005, and *P* = 0.009).

There were no significant centre differences on overall satisfaction scores and all subscales.

## 4. Discussion

This analysis of the psychological impact of using the automated closed-loop MD-Logic system under real-life conditions in the patients' home demonstrated reduced worries of hypoglycaemia with the artificial pancreas. Among children as well as adolescents and adult patients with T1DM alike there was a high level of satisfaction and increased acceptance of controlling nocturnal blood glucose automatically.

Hypoglycaemia, especially at night, is a major concern of patients and parents. It can impair well-being and is generally accepted as major obstacle to reach near-normoglycaemia [18, 19]. New technologies like CSII and CGM can improve glycemic control but still cannot solve the problem of nocturnal hypoglycaemia sufficiently [20]. Recent studies of our group and others with a night-time closed-loop system demonstrated that the closed-loop system is effective reducing the rate of nocturnal hypoglycaemia and increasing time within range in the home setting [5, 6, 21]. The present results confirm that these positive clinical results translate into positive psychological well-being with the MD-Logic system reducing worries of hypoglycaemia and increasing acceptance and satisfaction with this new technology in all age groups under real-life conditions.

It should be noted that fear of hypoglycaemia scores at study entry were already relatively low in all age groups but comparable to those reported in the literature for adults [22, 23], adolescents [24], and children [15]. This low HFS level had to be expected as patients with a particular history of severe hypoglycaemia were not included in the study for safety reasons. Nevertheless, despite the already low baseline level of HFS, a significant reduction in the Worry Subscale after using the AP system was found. This scale is known to reflect the cognitive level of fear of hypoglycaemia. Thus these findings may relate to AP patients experiencing less and even more reliable alarms compared to using sensor-augmented pump therapy (SAP). A significant reduction of hypoglycaemia and a lower rate of hypoglycaemia alarms during the closed loop nights with the MD-Logic AP versus control nights were demonstrated in the interim findings of the main study [5]. This may reinforce patients' trust in CLS and reduce their worries concerning episodes of hypoglycaemia. Our current findings are in contrast to another study on short-term use effects of CGM on fear of hypoglycaemia [25]. Without automated closed-loop insulin adjustment no reduction of fear of hypoglycaemia was observed with CGM alone. The authors argued that this finding may have been related to the low CGM accuracy and a high rate of false alarms. Adolescents especially reported frequent alarms as a barrier to using CGM continuously [26].

In our study the Behaviour Subscale of HFS remained unchanged on a low level. This can be explained due to the short time of the study. After only four nights it is unlikely that a major behaviour change can be observed. In another study after a two-month blood glucose awareness training, which focused on behavioural aspects, both scales were significantly reduced [27]. Currently 60-day studies with the MD-Logic under home conditions are underway. It will be interesting to analyse if such a longer period will eventually lead to changes in behavioural parameters. Nevertheless, our findings indicate an improvement in well-being in patients with T1DM using the MD-AP with less worries concerning episodes of hypoglycaemia and its consequences.

Satisfaction with the CLS has been assessed with a newly developed questionnaire to assess CL-satisfaction. The CL-satisfaction questionnaire demonstrated good internal reliability. In general, satisfaction with CLS has been relatively high in our study, with a mean score of about 3 (on a 0- to 4-point scale). CL-satisfaction was related to age, with lower satisfaction regarding “Ease of Use” of the AP in children than in adolescents and adults. This finding raises the issue that children (10–14 yrs) need the support and positive motivation of their caregivers for managing their diabetes tasks with the CLS. As a practical consequence, the developments of age appropriate education materials and specific curricula for children and their caregivers before starting the AP need to be implemented.

Despite the considerable technical prerequisites of using the CLS, the barriers of CLS in daily life were rated very low, especially in patients with regular CGM use. Potential hassles concerning the interpretation of a lot of data are considered a major barrier to CGM use. Therefore previous regular CGM experience at baseline may have given the patient sufficient knowledge to understand the more complex issues related to the CLS (e.g., sensor information and alarms). The CLS finally allows them to profit from the benefits of real-time CGM without the need for making sense of fluctuating glucose levels. Similar results were seen in a study comparing CGM before starting CSII versus CGM after using CSII. The group with CGM use before the start of CSII eventually turned out to use CGM more frequently [28]. A potential recommendation for future success with the CLS may be implementing a longer CGM experience prior to starting CLS.

The level of acceptance of CLS has been assessed with the adapted TAM questionnaire [12]. In general, acceptance of an AP has been relatively high in all age groups even before participants had any experience with the CLS overnight at home, with mean score of about 4 (on a 0 to 6 scale). After 4 nights with an AP participants reported significantly higher “Perceived Ease of Use” of the AP independently of age. Likewise the other acceptance scales remained on a high level. These findings demonstrate high acceptance before and after CL experience. Similar to the satisfaction results participants with regular sensor use reported significant higher acceptance scores of an AP than participants without regular sensor use. It can be summarized that patient satisfaction with and acceptance of the AP have been relatively high, and patients who used CGM regularly before starting AP reported higher satisfaction with and higher overall acceptance of an AP.

Recently a study was published regarding the psychosocial impact of overnight CLS at home for 15 adolescents with T1DM by Cambridge Group [13]. High satisfaction with the closed-loop system and a decrease of the mean HFS total score were reported to be similar to our data. However, inferential statistical analysis and comparison to our data were not possible due to the small sample size of the Cambridge Group.

The major strength of this study is that it provides evidence of the psychological effect of a CLS under real life conditions for different age groups. As the patients are asked to wear two devices (sensor and pump) as well as a laptop with the algorithm this high acceptance level of the system by patients is reassuring. Long-term adherence to CLS tasks will be necessary for the efficacy of this new technology. In CSII users with poor adherence to CSII tasks the efficacy of CSII in youth is limited [29]. Clearly CLS may reduce the burden of several diabetes tasks and could provide a significant benefit to the patients. They will be relieved from giving boluses, adjusting the basal rate or calculating insulin-to-carb ratios. Nevertheless, the patients' involvement in some of the diabetes management tasks will remain when using the CLS. They will still need to treat (rare) hypoglycaemia with carbohydrates, change the insulin catheter and sensor, or check the blood glucose for sensor calibration. Thus, in spite of the potential ease in diabetes management through the CLS, the human factor still needs to be taken into consideration.

The study covered a total period of 4 nights with the CLS. Important short-term effects of the MD-Logic AP on fear of hypoglycaemia and satisfaction with and acceptance of an AP were demonstrated. Several limitations of the present study have to be kept in mind. This study may not be adequately powered as the psychological aspects were not the primary end points. Also, the pediatric participants may not be able to provide all answers correctly. In a next step the psychological effect of an AP during long-term overnight and day-and-night use will be studied. Moreover, this study included only subjects without DKA or recent severe hypoglycaemia, but previous studies have shown that patients in poor metabolic control benefitted to a much greater extent from new technologies like SAP [30]. Thus it will be a future challenge to evaluate if the AP technology could also provide a significant step forward for subgroups with frequent acute diabetes complications.

## 5. Conclusions

In conclusion, this study demonstrated the positive psychological effect of an AP system in patient's home on fear of hypoglycaemia and satisfaction with and acceptance of an AP in children, adolescents, and adults with T1DM. By using the MD-Logic AP for four consecutive nights in home setting worries of hypoglycaemia were reduced in all age groups. In addition high satisfaction with and increasing acceptance of this new technology were reported after using the MD-Logic AP in home setting. This may predict an effective long-term use of the AP system by the patients in the future.

## Figures and Tables

**Table 1:** Baseline patient characteristics.

	Children (10–14 yrs) (*n* = 20)	Adolescents (14–18 yrs) (*n* = 20)	Adults (>18 yrs) (*n* = 19)	*P* value	Israel (*n* = 29)	Slovenia (*n* = 15)	Germany (*n* = 15)	*P* value	All (*n* = 59)
Age (years), mean (SD)	12.3 (1.17)	15.6 (0.86)	31.22 (9.96)		17.45 (6.43)	22.07 (12.00)	20.96 (12.96)	0.286	19.51 (9.98)

Male (%)	50	60	52.6	0.806	55.2	46.7	60.0	0.757	54

HbA_1c_ (%), mean (SD)	7.97 (0.72)	7.93 (0.76)	7.78 (0.58)	0.679	8.03 (0.71)	7.89 (0.74)	7.63 (0.53)	0.184	7.89 (0.69)

HbA_1c_ (mmol/mol, IFCC), mean (SD)	63.50 (7.90)	63.15 (8.33)	61.44 (6.32)		64.17 (7.78)	62.78 (8.13)	59.84 (5.75)		62.72 (7.51)

Diabetes duration (years), mean (SD)	7.25 (3.06)	8.78 (3.31)	19.24 (10.82)	0.000	9.21 (5.42)	13.92 (12.29)	14.03 (7.85)	0.094	11.63 (8.44)

CSII duration (years), mean (SD)	5.68 (3.35)	6.32 (2.82)	8.96 (5.58)	0.036	5.77 (3.52)	7.45 (3.24)	8.74 (5.70)	0.075	6.95 (4.24)

Regular sensor use (%)	65	55	26.3	0.044	62.1	53.3	20.0	0.028	49.2

**Table 2:** Acceptance of an artificial pancreas analysis.

		Pre	Post	Delta	*P* value
Total acceptance	All	4.69 (0.87)	4.76 (1.06)	0.07 (0.77)	0.501
Intention to Use	All	4.7 (1.25)	4.76 (1.64)	0.01 (1.44)	0.964
Perceived Usefulness	All	4.66 (0.91)	4.67 (1.07)	0.01 (0.86)	0.940
Perceived Ease of Use	All	4.64 (0.94)	5.06 (1.09)	0.42 (0.95)	0.002

Total acceptance	Children (*n* = 17)	4.54 (1.04)	4.54 (1.15)	−0.00 (0.67)	
	Adolescents (*n* = 19)	4.69 (0.77)	4.88 (1.18)	0.19 (0.86)	
	Adults (*n* = 19)	4.81 (0.82)	4.83 (0.86)	0.01 (0.80)	

Values are expressed as mean (SD).

**Table 3:** Association between acceptance of an artificial pancreas and regular sensor use initial.

		Regular sensor use	Regular sensor use	*P* value
		Yes	No	
Total acceptance	All	5.07 (0.59), *26 *	4.34 (0.93), *29 *	0.000
Intention to Use	All	5.20 (1.01), *28 *	4.33 (1.32), *30 *	0.007
Perceived Usefulness	All	5.02 (0.67), *27 *	4.34 (0.98), *30 *	0.004
Perceived Ease of Use	All	5.03 (0.77), *25 *	4.28 (0.96), *27 *	0.004

Total acceptance	Children	5.05 (0.81), *10 *	3.80 (0.90), *7 *	
	Adolescents	5.09 (0.42), *11 *	4.14 (0.81), *8 *	
	Adults	5.07 (0.49), *5 *	4.72 (0.90), *14 *	

Values are expressed as mean (SD), *n*.

**Table 4:** Satisfaction scores after 4 nights with the MD-Logic artificial pancreas in home setting.

	Children	Adolescents	Adults	*P* value
Total satisfaction	2.96 (0.52)	3.06 (0.60)	3.04 (0.53)	0.848
Perceived Usefulness of Alarms	2.84 (0.88)	2.74 (0.80)	2.87 (0.64)	0.872
Trust	2.95 (0.70)	3.22 (0.80)	3.07 (0.88)	0.600
Ease of Use	2.85 (0.83)	3.35 (0.60)	3.60 (0.55)	0.004
Satisfaction	3.15 (0.69)	3.25 (0.91)	3.07 (0.75)	0.804
Freedom	3.03 (0.66)	2.58 (0.95)	2.37 (1.01)	0.068

Values are expressed as mean (SD).

## Abbreviations

AP: Artificial pancreas
CGM: Continuous glucose monitoring
CLS: Closed-loop system
CSII: Continuous subcutaneous insulin infusion
DREAM: Diabetes Wireless Artificial Pancreas Consortium
HFS: Hypoglycaemia Fear Survey
SAP: Sensor-augmented pump therapy
TAM: Technology Acceptance Model
T1DM: Type 1 diabetes mellitus.
